# Winter locations of red‐throated divers from geolocation and feather isotope signatures

**DOI:** 10.1002/ece3.9209

**Published:** 2022-08-23

**Authors:** James Duckworth, Susan O'Brien, Ib K. Petersen, Aevar Petersen, Guðmundur Benediktsson, Logan Johnson, Petteri Lehikoinen, David Okill, Roni Väisänen, Jim Williams, Stuart Williams, Francis Daunt, Jonathan A. Green

**Affiliations:** ^1^ University of Liverpool Liverpool UK; ^2^ Scottish Government, Marine Laboratory Aberdeen UK; ^3^ Department of Bioscience Aarhus University Aarhus Denmark; ^4^ Independent researcher Reykjavik Iceland; ^5^ Independent researcher Shetland Scotland; ^6^ Finnish Museum of Natural History University of Helsinki Helsinki Finland; ^7^ Avescapes Oy Helsinki Finland; ^8^ Independent researcher Orkney Scotland; ^9^ UK Centre for Ecology & Hydrology Penicuik Midlothian UK

**Keywords:** *Gavia*, GLS, isotope, loon, movement

## Abstract

Migratory species have geographically separate distributions during their annual cycle, and these areas can vary between populations and individuals. This can lead to differential stress levels being experienced across a species range. Gathering information on the areas used during the annual cycle of red‐throated divers (RTDs; *Gavia stellata*) has become an increasingly pressing issue, as they are a species of concern when considering the effects of disturbance from offshore wind farms and the associated ship traffic. Here, we use light‐based geolocator tags, deployed during the summer breeding season, to determine the non‐breeding winter location of RTDs from breeding locations in Scotland, Finland, and Iceland. We also use δ^15^N and δ^13^C isotope signatures, from feather samples, to link population‐level differences in areas used in the molt period to population‐level differences in isotope signatures. We found from geolocator data that RTDs from the three different breeding locations did not overlap in their winter distributions. Differences in isotope signatures suggested this spatial separation was also evident in the molting period, when geolocation data were unavailable. We also found that of the three populations, RTDs breeding in Iceland moved the shortest distance from their breeding grounds to their wintering grounds. In contrast, RTDs breeding in Finland moved the furthest, with a westward migration from the Baltic into the southern North Sea. Overall, these results suggest that RTDs breeding in Finland are likely to encounter anthropogenic activity during the winter period, where they currently overlap with areas of future planned developments. Icelandic and Scottish birds are less likely to be affected, due to less ship activity and few or no offshore wind farms in their wintering distributions. We also demonstrate that separating the three populations isotopically is possible and suggest further work to allocate breeding individuals to wintering areas based solely on feather samples.

## Graphical abstract

Understanding the distributions of species vulnerable to disturbance from anthropogenic activity is vital in assessing future consequences. Using remote sensing technology, we tracked the movements of red‐throated divers from three breeding locations to identify their overwinter distributions. We then linked these population level differences in distribution to differences in isotopic signatures of feathers grown during the moulting period.

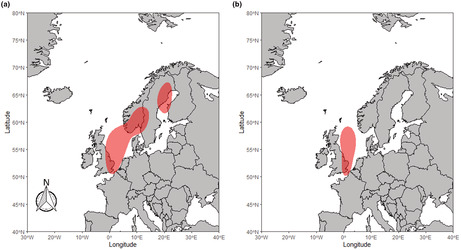

## 1 INTRODUCTION

Identifying the migratory strategy and wintering locations of populations and connecting them to the relevant breeding grounds allows for more effective strategies of management and if necessary, conservation (Strøm et al., 2021). Furthermore, the migratory strategy adopted by a population will dictate the geographic area occupied; and therefore, influence the environmental conditions it must withstand during the non‐breeding period. As a result, conditions faced by different populations across a species' range can be vastly different, which can, in turn, lead to variation in demographic rates both during the season in question and in the subsequent season through carry‐over effects (Frederiksen et al., 2012). For example, carry‐over effects from the non‐breeding season may cause reduced breeding success due to stress in wintering ground habitat quality (Fayet et al., 2016). However, investigating these processes is challenging during the non‐breeding period, particularly for populations that become largely inaccessible due to them solely using marine habitats. Therefore, studies which overcome this difficulty provide a valuable and unique insight into a poorly known period of the annual cycle. This knowledge is all the more pressing in species where negative interactions with future anthropogenic stresses, such as offshore wind farms, are predicted (Dierschke et al., 2016).

For some species of bird, many of the detrimental effects from windfarm developments likely occur specifically during the molt and winter period (Dierschke et al., 2017; Heinänen et al., 2020). Some diving birds, including divers (or “loons”; *Gavia* spp), undergo a synchronous molt of their flight feathers, rendering them flightless for a few weeks (HiDEF, 2016; Kjellén, 1994). During molt, a combination of a reduced ability to relocate and the high energetic costs of molt, make them particularly vulnerable to anthropogenic effects (Buckingham, Bogdanova, et al., 2022). Therefore, linking the molting and winter distributions to the associated breeding population is essential in quantifying the potentially deleterious effects of offshore wind farm interactions on demographic rates, such as survival or breeding success. Red‐throated divers (RTDs; *Gavia stellata*) are one such species and have recently been the focus of much interest due to their avoidance of offshore windfarms and associated activity (Furness et al., 2013; Heinänen et al., 2020). One of the most pressing knowledge gaps currently is understanding the molting and winter distributions used by different breeding populations. This knowledge will enable subsequent research and monitoring to ensure effects of perturbations in the key periods of molt, and midwinter can be attributed to the correct breeding populations, to quantify both influences during the non‐breeding season and carry‐over effects into the breeding period (Harrison et al., 2011).

Studies in North America have shown RTD moving between continents, with movements up to 8000 km from breeding grounds in Alaska to wintering areas in Asia and along the Pacific (McCloskey et al., 2018). In Europe, birds from many populations can make large migratory flights, while some are thought to fly short distances or remain resident (Dorsch et al., 2019; Duckworth et al., 2020). Furthermore, year‐round variation in habitat use can differ between individuals and populations, with RTD switching from a marine to a wholly freshwater distribution from the non‐breeding to the breeding season, respectively (Duckworth et al., 2021). However, in Europe, we currently lack a comprehensive understanding of the year‐round distributions of all populations. Therefore, to understand the environmental pressures individuals and populations face, we must first identify the areas that different breeding populations occupy in the non‐breeding period. Historically, bird band recoveries have been used to gather information that links breeding and non‐breeding season locations of RTDs, for example, birds breeding in Scotland have been recovered in the Southern North Sea and around Scotland, suggesting a partial migration strategy (Okill, 1994). However, these methods generally only provide information on birds, which have perished and may be biased toward revealing unsuccessful strategies (Bairlein, 2001). Currently, the best methods to determine migratory movements of seabirds are through the deployment of biologging devices (Laurenson et al., 2021), of which leg‐mounted light‐based geolocators are often the smallest and least intrusive device (Bodey et al., 2018).

While biologging has revolutionized our understanding of avian migration (Fudickar et al., 2021), where possible, attempts should be made to develop methods to determine the distributions of birds of an unknown origin, without the need for any potentially invasive deployments. This is particularly relevant to divers, which are vulnerable to disturbance and stress by human interventions (O'Brien et al., 2020; Rizzolo et al., 2014). Isotope analysis has the ability to provide a wide range of insight into the diet (Hobson et al., 1994; Weiss et al., 2009), behavior (Votier et al., 2011) and movement (St John Glew et al., 2018) of many marine species. Working to build an understanding of the isotopic differences within and between populations of a species has the potential to inform methods for less invasive identification of migratory behaviors (Jaeger et al., 2010). For example, previous work by St John Glew et al. (2018) to understand the locations of wintering guillemots using feathers grown during their annual molt has allowed for the broad determination of molt location in the North Sea from a combination of δ^15^N and δ^13^C, isotope signatures in feather samples using an isoscape. The full development of such methodologies requires calibration using data on environment and location of the population along with a suitable habitat‐based isoscape covering the relevant area (Carpenter‐Kling et al., 2020). The principal metric required for this work is an enrichment factor, representing the difference in isotope values between the study organism and organism the isoscape was built with, driven by differences in both the prey and trophic level the organisms consume. While such information is not currently available for RTDs, beginning to link distribution to isotope values in RTDs will undoubtedly have a role in developing future methodologies for movement patterns in this species. Furthermore, isotope data retrieved from feathers will provide information over the time period they were grown, which in RTD is during the autumn equinox. During the equinox periods, GLS data are less reliable, as the differences in day length, the metric used to determine latitude, across latitudes becomes near uniform globally. Therefore, isotope approaches can be used to provide information on distribution when GLS data are potentially unviable due to the equinox (Lisovski et al., 2012).

In this study, we aim to present the first biologging and isotope data on locations used by RTDs from three populations in NW Europe during the winter non‐breeding season and describe the migration strategies of each of the populations. To achieve this, we deployed light‐based geolocators to show the distribution of the birds during the winter. We also plucked feathers from RTDs during recapture events. These feathers were used to reveal the differences in isotope signatures between the three populations during the molt period, through stable isotope analysis. Through combining these two data streams, we provide results on the distribution of the populations during the non‐breeding period and explore whether future work could identify non‐breeding distributions of individuals using only feather isotopes.

## 2 METHODS

From May to July in 2018–2019, 89 (Finland *n* = 32; Scotland *n* = 38; North Eastern Iceland *n* = 19) RTDs were captured using a combination of nest traps and extended mist nets (O'Brien et al., 2020) and equipped with GLS tags (Biotrack/Lotek MK4083 Geolocator) on a plastic leg ring. Fifty‐four of the deployed tags were recovered and removed 1–3 years after deployment, each with 1–2 years of data (Thompson et al., 2022). All birds were handled for <10 min, and if any sign of skin damage was observed, the bird was not retagged. In total, sufficient data on the wintering periods (defined as where the GLS functioned until at least December) were obtained for 8, 8, and 11 individuals (from 11, 8, and 13 retrieved functioning GLS tags) from Finland, Scotland, and Iceland, respectively, including individuals where tags were deployed twice. Seventy‐six secondary flight and 64 secondary covert feather samples were taken for isotopic analysis from birds in the 2019 and 2020 field seasons. These samples corresponded to the molt period from September to October (Dorsch et al., 2019) in 2018 and 2019. GLS tags were also deployed from 2007 to 2012 in Scotland and Western Iceland in earlier studies, using the same methods as above, with six and five functioning GLS tags recovered in subsequent field seasons from Scotland (Shetland only) and Western Iceland, respectively. No feathers were taken during this earlier study period. Here, we present all data from the non‐breeding period obtained by GLS tags.

Two locations per day were generated from the GLS data using the BASTrack collection of software packages. Following initial observations of light levels during twilight events and values suggested by the software instructions, a light threshold value of 15 was used to determine sunset and sunrise. Across individuals, this light threshold value was related to a mean sun elevation angle of −5. No other post‐processing or landmask was used to generate locations. Population‐level estimates of core distributions used were estimated from the 50% kernel density contour, which has been shown to provide the best estimate for location estimates of populations when considering GLS errors (Buckingham, Daunt, et al., 2022). These were generated using the adehabitatHR R package (Calenge, 2006), with the “href” function used to generate the smoothing parameter, with the grid size set to 1000 and an extent of 1. All available locations from all individuals within the stated timeframes are used to generate estimates. RTDs from our study populations completed their breeding attempts by mid‐late August (Duckworth et al., 2021), but locations shown are from the early winter period (22nd October–31st December) to late winter period (1st January–20th February) to exclude periods where there is still a noticeable impact on locations from the equinox periods. To further exclude any clearly anomalous data points, any points above 75° North were excluded, as often locations extracted when the GLS logger is heavily shaded are pushed to the northernmost degrees of latitude. To ensure kernel distributions for populations were not biased toward individuals with more years of data, an average location for each calendar date was taken for those individuals across the study period. This meant each individual had equal weighting in the final population kernel. This averaging is justified by the high repeatability of movements of individuals between years seen within our study and others on divers (Dorsch et al., 2019; Paruk et al., 2015).

Feathers were stored in paper envelopes at room temperature for 4 months prior to isotope analysis, which was carried out by Elemtex Ltd. Samples were washed 3 times in a solution of 2:1 chloroform and methanol and rinsed in distilled water, before being oven‐dried at 60°C. Subsequently, the samples were run on an ANCA/2020 isotope ratio mass spectrometer, which was set to run in continuous flow mode. Finally, data were normalized to Vienna PeeDee Belemnite for δ13C and Air for δ15N using USGS40 and USGS41A as reference materials (Qi et al., 2016), with typical precisions being better than 0.3 ml^−1^. Isotope values are expressed as δ^15^N and δ^13^C, which represent the relative difference, in parts per thousand, of the ^15^N and ^13^C isotopes, relative to their respective standard.

To determine whether isotope values and hence locations used during molt were distinct across the three sampled locations, linear discriminant analysis (LDA) was applied to the δ^15^N and δ^13^C signatures of all feathers (regardless of retrieved from birds caught during the 2019 and 2020 field season). A model was generated with LDA separately for isotope signatures from the secondary covert and secondary flight feathers to determine whether either of the feathers is better able to separate the populations. If a successful LDA model could be created with either secondary flight or covert feathers, it would mean only covert feathers would need to be sampled for future isotope work, which is thought to be less disruptive to the birds. Training of the LDA models was carried out with a subset of 80% of the available data, and testing was carried out with the remaining 20% to verify the classifications.

## 3 RESULTS

**FIGURE 1:**
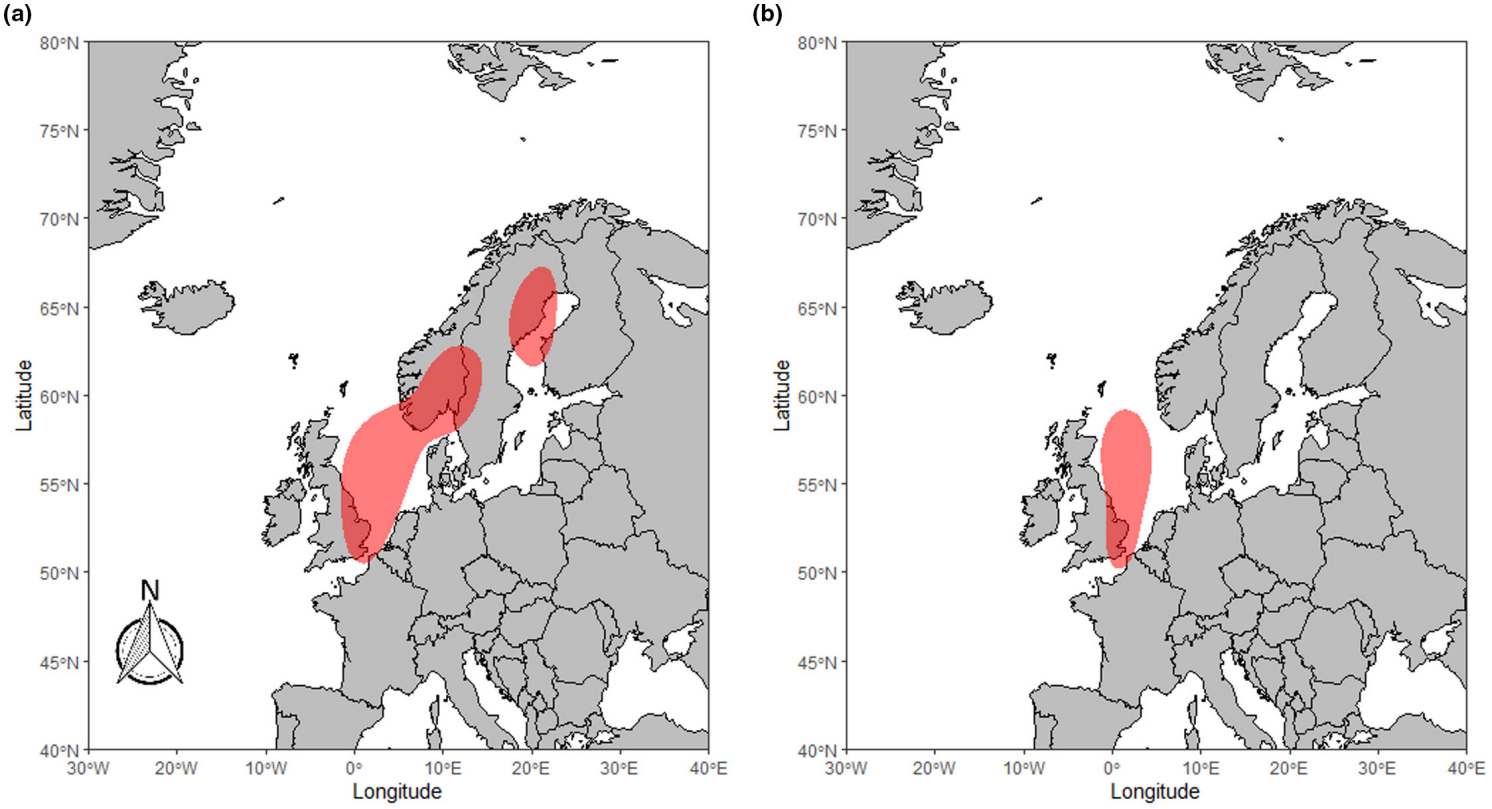
50% kernel density distribution of the locations of RTDs sampled in Finland during the early (a) and late (b) winter period. Both panels show the 2017–2021 study period.

**FIGURE 2:**
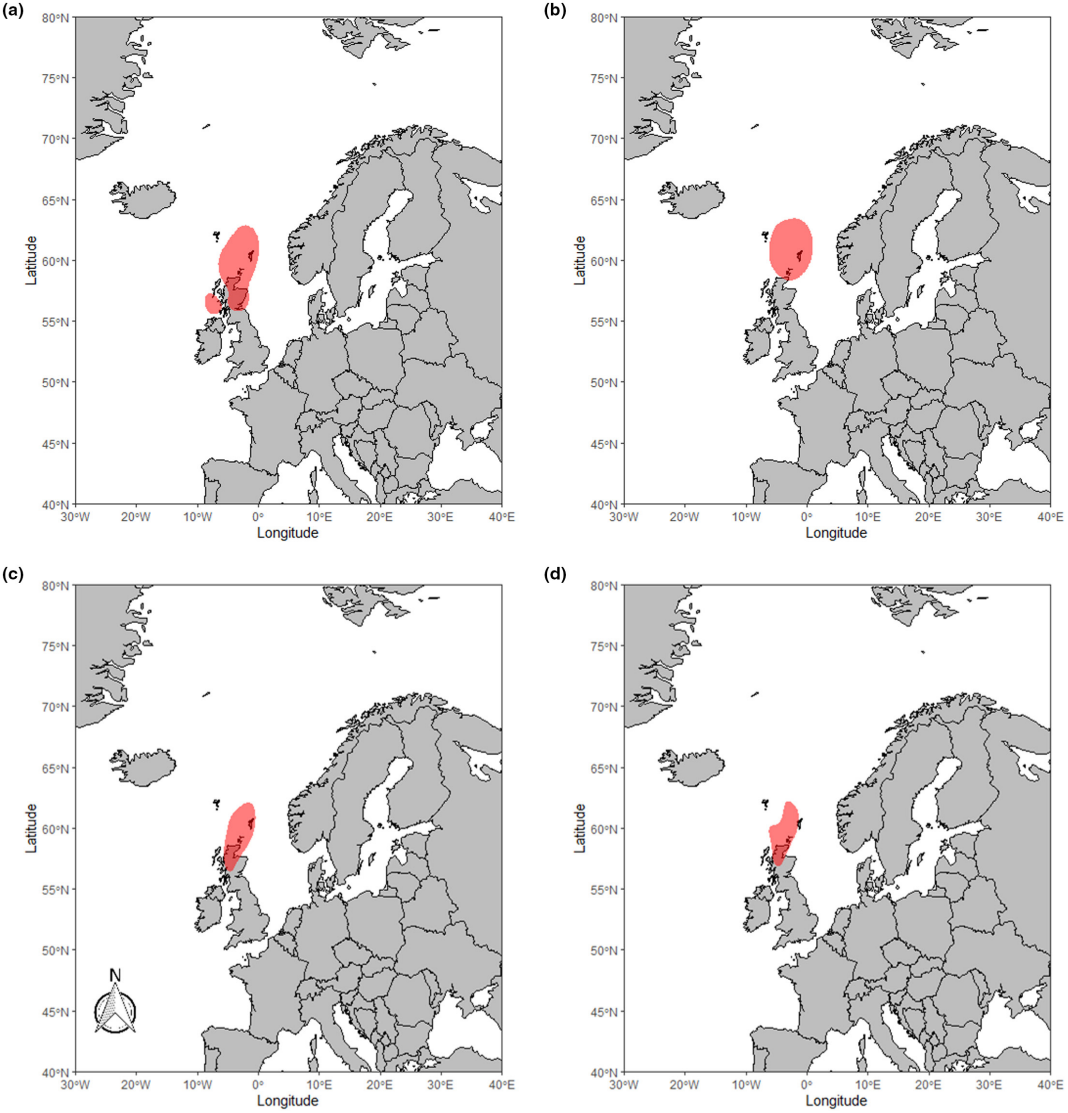
50% kernel density distribution of the locations of RTDs sampled in Scotland during the early (a,c) and late (b,d) winter period. Panels (a,b) show the 2017 to 2021 study period, while (c,d) show the 2007–2010 study period.

**FIGURE 3:**
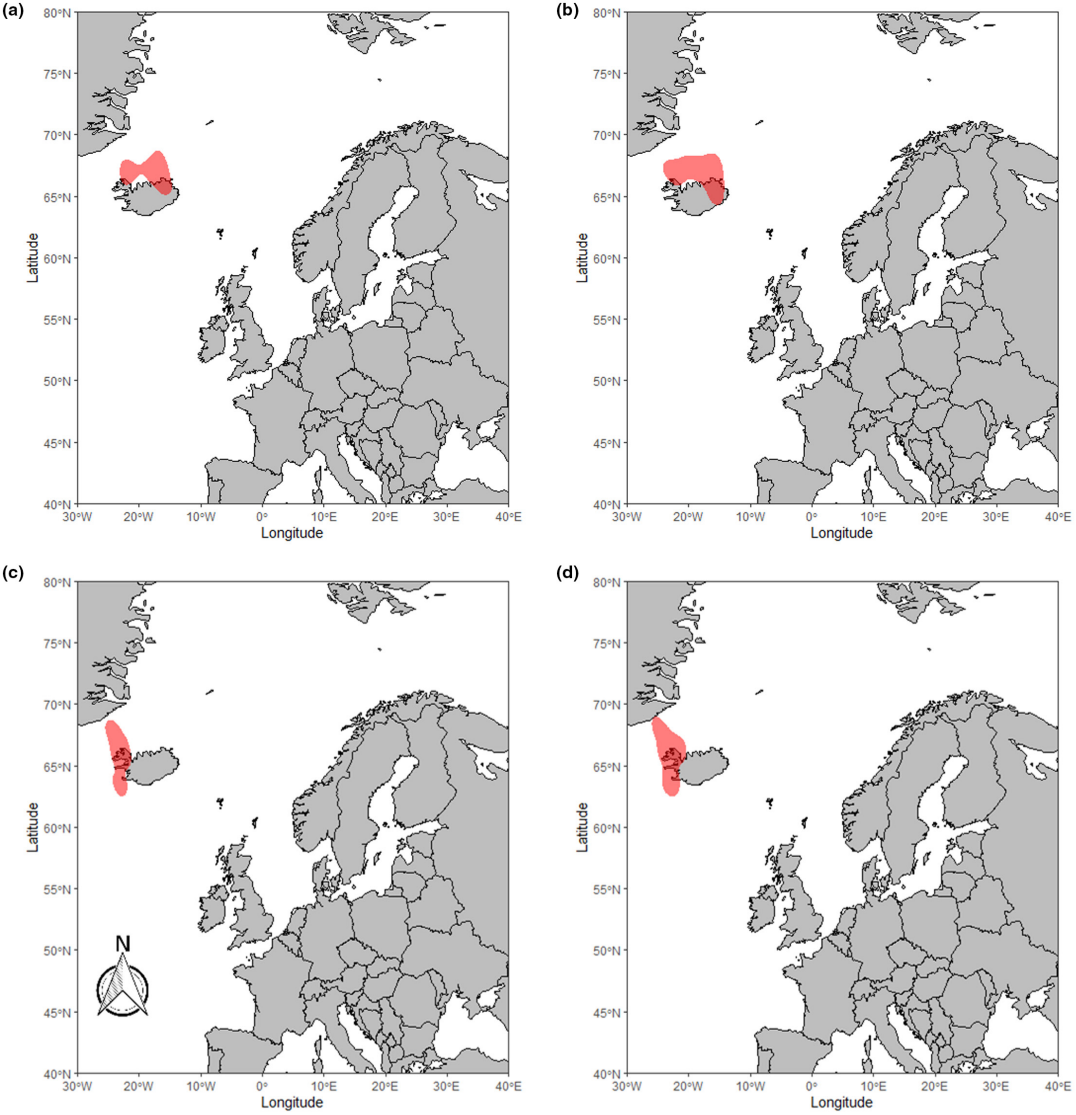
50% kernel density distribution of the locations of RTDs sampled in Iceland during the early (a,c) and late (b,d) winter period. Panels (a,b) show the 2017–2021 study period in East Iceland, while (c,d) show the 2007–2010 study period in West Iceland.

RTDs from Finland migrated westwards from their breeding grounds in mainland Finland through to the western Baltic in the early winter (Figure 1a) and southern North Sea by late winter (Figure 1b). Birds from Scotland in both study periods showed a varied pattern of movement (Figure 2a,c), with some birds remaining around the northern Scottish isles. In contrast, others migrated a short distance to other coastlines around northern mainland Britain and Northern Ireland. In the later winter period for Scotland RTDs (Figure 2b,d), the 50% kernel indicated much of the core area is on land in Scotland. This may be the result of some individuals making movements southwards along either the East or West coasts of the British Isles, as well as some of the GLS tags failing before the late winter period. These results suggest RTD from the Scotland population on Orkney and Shetland can be thought of as partial migrants, with some individuals remaining resident and others migrating for at least some of the period. Birds from both East Iceland (Figure 3a,b) and West Iceland (Figure 3c,d) were resident year‐round, largely remaining around the northern coast of Iceland throughout the winter, only moving distances over 200 km from their breeding season locations in a few cases (Figure 3). These small movements were largely longitudinal; therefore, we can conclude this was likely due to movement rather than GLS errors. While our sample size was not sufficient to formally investigate inter‐annual consistency in wintering grounds, all individuals sampled across multiple years showed consistency in sites used in the winter.

**FIGURE 4:**
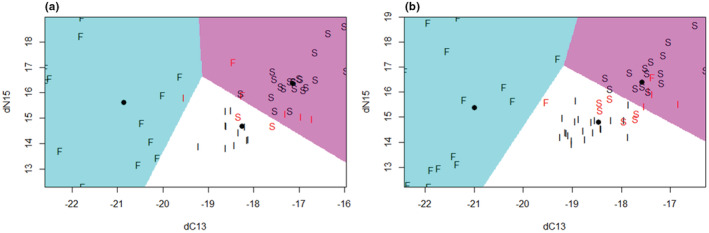
Outputs of the linear discriminant analysis. Data shown here are from the training partition of the overall dataset. Letters represent the population an individual data point was sampled from. Letters in red represent points from the training dataset that were misclassified. Shaded areas represent the population within which a point would be classified as originating from Finland, Iceland, or Scotland as blue, white, or pink, respectively. (a) Shows the model for the secondary flight feather, and (b) shows the model for secondary covert feathers.

LDA models created with the isotope data were both able to separate the populations based on the isotope signatures. The models had an accuracy of 91% and 86% for secondary flight feathers and secondary covert feathers, respectively, when applied to the testing datasets (Table 1). The outputs of the two LDA models are visualized in Figure 4 to show boundaries of the classification regions. Table 2 shows that in terms of population average, the differences between the two feather types are small. In the case of both feather types, linear discriminant (LD) 1 is strongly associated with δ^13^C and LD 1 subsequently contributes greater than 90% of the trace in both models, suggesting δ^13^C is the more important isotope when looking at spatial separation (Table 1). Figure 4 demonstrates this importance with the majority of variation being shown across the δ^13^C axis and variation in δ^15^N mostly occurring within sites, especially in Finland.

**TABLE 1:** Results of the linear discriminant analysis showing the loadings of δ^15^N and δ^13^C onto the linear discriminant axes for models generated from the secondary covert and secondary flight feathers. Model accuracy gives the proportion of correctly identified country of origins of the test data predicted by the model built from the training data.

Feather	Model accuracy	Coefficients of linear discriminant 1		Coefficients of linear discriminant 2		Proportion of trace for linear discriminants	
		δ^15^N	δ^13^C	δ^15^N	δ^13^C	1	2
Flight	0.909	0.009	1.05	0.810	−0.131	0.902	0.098
Covert	0.857	−0.265	1.258	0.768	−0.107	0.9137	0.0863

**TABLE 2:** Group means and standard deviations from the secondary flight and secondary covert LDA models for their δ^13^C and δ^15^N signatures

Feather	Finland		Iceland		Scotland	
	δ15N	δ13C	δ15N	δ13C	δ15N	δ13C
Flight	15.62 (1.96)	−20.87 (1.44)	14.68 (0.60)	−18.26 (0.76)	16.38 (0.91)	−17.15 (0.59)
Covert	15.39 (2.29)	−21.00 (1.34)	14.80 (0.60)	−18.47 (0.67)	16.41 (0.99)	−17.59 (0.58)

## 4 DISCUSSION

Both the GLS and isotope data lend support to the three populations of RTDs spending the non‐breeding season in spatially separated locations and demonstrating three different migratory strategies. The results from GLS devices suggest the RTDs from Iceland remain resident around Iceland, with only movements around the coasts of Iceland being observed. RTDs from Scotland (Shetland and Orkney) are shown to either remain resident, move to the coastal waters of the Western Isles, or make movements south to the coasts of mainland Scotland and Northern Ireland. However, during the late winter period, we no longer see the separate distributions for the migrants and residents with only the resident distribution remaining. This is likely due to logger failure within the population. Previous studies have demonstrated that individuals from Scotland have migrated and likely remained in these areas beyond the early winter (Okill, 1994). Therefore, this evidence suggests RTDs from Scotland are partial migrants. RTDs from Finland have the longest migration distance of the three populations, and the population kernel was shown to move westward as the season progressed (Figure 1), indicative of a fully migratory strategy. This population was shown to move from the eastern Baltic Sea, likely molting in this area, through to the western Baltic, southern North Sea and east coast of England. Our isotope results corroborate these findings, in that the three have distinct isotopic signatures from the molt period (assumed to occur in September–October) with the differences in δ^15^N and δ^13^C values across the three populations indicating the use of different locations at this time. However, there is likely only a small temporal overlap in our GLS locations and isotope results, as the equinox precludes the inclusion of GLS data during part of the molt period (September to early October). Therefore, the results in tandem provide evidence of complete segregation of populations from the start of molt, through to the end of the wintering period.

The limited amount of movement observed in the Icelandic birds likely means molt is occurring in similar locations to the rest of the non‐breeding period locations. This population does still experience some seasonal change though, as they cease spending time in freshwater environments during the non‐breeding season (Duckworth et al., 2021). Our maps showed a westward movement for some birds during the study period (Figure 3a,b), from the northeast to northwest coast of Iceland. This suggests there may be short movements for some individuals. In contrast, others, including those from the earlier deployments (Figure 3c,d), remain at locations indistinguishable by GLS tags from their breeding season locations in most scenarios. Scottish RTDs which do not leave the waters surrounding Shetland and Orkney will likely molt in these areas. However, it is unclear whether the Scottish RTDs that are migratory, moving to the coasts around either mainland Great Britain or Ireland, molt before or after departure from their breeding grounds.

This study used stable isotope data to demonstrate the separation of three populations during the molt period and suggests further work could apportion breeding individuals to molting locations based solely on feather samples. We found the δ^13^C signatures of the three populations separate into distinct clusters, along with δ^15^N to a lesser degree (Figure 4 and Table 1). These results suggest that a method to identify the molting grounds of individual birds based on feather samples and isotope analysis is possible, like those created in other study systems (Cruz‐Flores et al., 2018; Jaeger et al., 2010). Further refinement of the methodology requires additional research to identify a wider range of molting areas from feather isotopes to establish the most commonly used molting locations by RTDs in Europe and their associated isotopic signature. However, in its current form, these results allow for the identification of individuals carrying out novel migration movements, as would be suggested by deviations from the population‐specific mean isotope signature detected in this study. In this regard, a better understanding of a wider array of locations used during molt in NW Europe could help identify movements across the metapopulation range.

The mean observed δ^15^N and δ^13^C values from the Finnish RTDs aligned with isotope signatures found by Dorsch et al. (2019) among RTDs molting in the Eastern Baltic (Figure 4). The locations we estimate our Finland RTD population to be in during the end of the molting period (mid‐late October) also line up with their locations for birds from other breeding populations, like Siberia, identified to molt in the Baltic Sea (Dorsch et al., 2019) (Figure 1). This provides us with a degree of confidence that any RTDs molting in the Baltic Sea will lie within the “Isotopic area” identified by our LDA. However, it is not possible to determine with a high degree of accuracy where exactly RTDs were molting within the Baltic Sea with the current isotope and location data. The cluster of individuals with a δ^13^C of −22 could be moving further North in the Baltic, as this area is known to produce more negative δ^13^C than the Southern areas of the Baltic Sea (Magozzi et al., 2017). Movement data with a high accuracy, like GPS, along with feather samples grown during the tracked period, would allow for investigation into whether a higher temporal resolution of isotope location differentiation is possible (Votier et al., 2011).

The accuracy of the two LDA models suggests using secondary flight feathers is preferable to secondary covert feathers when trying to separate the three populations (Table 1 and Figure 4). The two feather types performed differently could be due to the specific timing and duration of the molt on the feather type. In terms of impact, it is worth noting that the removal of part of a secondary flight feather is likely to be greater on aerodynamics and effort required for flight, compared with a covert feather. However, other studies have sampled primary flight feathers (White & Dawson, 2021; Yerkes et al., 2008), so our approach of using a small section of secondary flight feathers over primaries should be seen as cautious. We believe future effort should be invested into further developing feather isotope maps with secondary flight feathers, as the slight increase in accuracy may be enough to help distinguish sites with smaller spatial differences, for a small trade‐off in disturbance. Additionally, RTDs are large birds, and only a small section of a secondary flight feather is needed to assess isotopic signatures, meaning the effects of removing this small section are likely to be minimal.

Another observation in this study is within‐population variation of isotope signatures was greater for RTDs from Finland than the other two populations, with Finland demonstrating a much higher range of δ^15^N values (Figure 4). This high variability is unlikely to be driven by location and movements alone, as δ^15^N does not vary as much spatially as δ^13^C and RTD are constrained in their movements at their molting areas, due to their inability to fly (Ceia et al., 2018; Gómez et al., 2018). The variation could be driven by variation in diet, habitat use or an isotopically diverse local environment (Dorsch et al., 2019; Duckworth et al., 2020). This could be a product of individual RTD selecting for different prey species within the same area, leading to the patterns we observed in δ^15^N signatures being driven by either benthic/pelagic or trophic level differences of prey. RTDs are generalist foragers (Kleinschmidt et al., 2019); therefore, a wide range of δ^15^N signatures is expected as RTD distribute their foraging efforts across a wider range of prey species. Cementing this relationship would require future work to link foraging behavior metrics on dive depth and behavior to δ^15^N isotope signatures.

Through the processing of GLS tags in our work, this study also flags the difficulties of using GLS tags with RTDs. Primarily, the interference to light levels experienced through various resting behaviors, such as leg tucking while resting is a significant disadvantage. Such shading will affect the reliability of latitudinal estimates, tending to drag locations of these northern hemisphere birds toward the north, but if tucking occurs at both the sunset and sunrise equally it is unlikely to affect estimates of longitude. Other studies have noted the effects of sensor shading on the accuracy of light‐based geolocation and deployed methods to remedy the issue (Bindoff et al., 2018; Merkel et al., 2016). However, shading of the sensors was so pronounced that even these methods failed to produce realistic estimates of locations and movements. For this reason, we used a simple method that applies no post‐processing adjustments or landmasks to generate locations. These findings are very much in line with recent work by Halpin et al. (2021), who suggest the location errors of 186 and 202 km for GLS are not uniform across species. We, therefore, strongly recommend that future studies aimed at exploring detailed locations of RTDs avoid the use of GLS. However, for research questions focused on establishing the type and scale of migration or only requiring data from the immersion sensor, GLS is a valid tool. This is compounded by the relatively low recovery rates of GLS loggers from the RTDs in our study, which was driven by a combination of inaccessible field sites, aversion of birds to human presence and Covid‐19 restrictions. Despite the errors in our individual location fixes, our overall population‐based location estimates give a good indication of the areas used during the non‐breeding season and hence migration strategy of RTDs from three NW European populations. Furthermore, the areas shown here overlap with the current understanding of distributions of RTDs during winter (Heinänen et al., 2020; Kleinschmidt et al., 2019; O'Brien et al., 2012) providing reassurance that the distributions presented here are reliable. Subsequent work should build on these results by continuing to link isotopes to locations, as the importance of developing a robust and low‐impact method for apportioning individuals to molting locations cannot be overstated.

Our results have shed light on molt and winter distributions of RTDs and demonstrated the different migration strategies across populations. Isotope signatures shown here have demonstrated differences between the three populations and hinted that future methods, such as the use of isoscapes, to determine molt locations of an individual of an unknown origin might be possible. This study has also helped emphasize that future work is needed to address the spatial and temporal extent to which different populations of RTDs might come into contact with anthropogenic activity. Importantly, our work suggests that populations from Iceland and Scotland may be less affected by offshore wind farm developments, as we found little evidence of movements to current areas of development. Conversely, RTDs from the Finland population are shown to move into areas of current and future development, specifically in the south North Sea.

## AUTHOR CONTRIBUTIONS

**James Alexander Duckworth:** Conceptualization (equal); formal analysis (lead); investigation (equal); methodology (lead); visualization (lead); writing – original draft (lead); writing – review and editing (lead). **Susan O'Brien:** Conceptualization (equal); funding acquisition (lead); investigation (equal); methodology (equal); project administration (equal); resources (equal); supervision (supporting); writing – review and editing (equal). **Ib Krag Petersen:** Conceptualization (equal); data curation (equal); funding acquisition (equal); investigation (equal); methodology (equal); project administration (equal); supervision (supporting); writing – review and editing (equal). **Aevar Petersen:** Conceptualization (equal); data curation (equal); funding acquisition (equal); investigation (equal); methodology (equal); project administration (equal); writing – review and editing (equal). **Guðmundur Benediktsson:** Methodology (equal); writing – review and editing (equal). **Logan Johnson:** Methodology (equal); writing – review and editing (equal). **Petteri Lehikoinen:** Methodology (equal); writing – review and editing (equal). **David Okill:** Methodology (equal); writing – review and editing (equal). **Roni Väisänen:** Methodology (equal); writing – review and editing (equal). **Jim Williams:** Methodology (equal); writing – review and editing (equal). **Stuart Williams:** Methodology (equal); writing – review and editing (equal). **Francis Daunt:** Conceptualization (equal); methodology (equal); supervision (supporting); writing – review and editing (equal). **Jonathan A Green:** Conceptualization (equal); funding acquisition (equal); investigation (equal); methodology (equal); project administration (equal); supervision (lead); writing – original draft (equal); writing – review and editing (lead).

## CONFLICT OF INTEREST

All authors declare no conflict of interest.

## Data Availability

Data will be stored on the publicly accessible repository managed by the JNCC at: https://hub.jncc.gov.uk/. Further inquiries can be made to data@jncc.gov.uk.
